# A Rapid Method to Characterize Mouse IgG Antibodies and Isolate Native Antigen Binding IgG B Cell Hybridomas

**DOI:** 10.1371/journal.pone.0136613

**Published:** 2015-08-28

**Authors:** Haolin Liu, Janice White, Frances Crawford, Niyun Jin, Xiangwu Ju, Kangtai Liu, Chengyu Jiang, Philippa Marrack, Gongyi Zhang, John W. Kappler

**Affiliations:** 1 Howard Hughes Medical Institute, Denver, Colorado, United States of America; 2 Department of Biomedical Research, National Jewish Health, Denver, Colorado, United States of America; 3 Department of Immunology and Microbiology, University of Colorado Denver, Colorado, United States of America; 4 State Key Laboratory of Medical Molecular Biology, Institute of Basic Medical Sciences, Chinese Academy of Medical Sciences, Beijing, China; Imperial College London, UNITED KINGDOM

## Abstract

B cell hybridomas are an important source of monoclonal antibodies. In this paper, we developed a high-throughput method to characterize mouse IgG antibodies using surface plasmon resonance technology. This assay rapidly determines their sub-isotypes, whether they bind native antigen and their approximate affinities for the antigen using only 50 μl of hybridoma cell culture supernatant. Moreover, we found that mouse hybridomas secreting IgG antibodies also have membrane form IgG expression without Igα. Based on this surface IgG, we used flow cytometry to isolate rare γ2a isotype switched variants from a γ2b antibody secreting hybridoma cell line. Also, we used fluorescent antigen to single cell sort antigen binding hybridoma cells from bulk mixture of fused hybridoma cells instead of the traditional multi-microwell plate screening and limiting dilution sub-cloning thus saving time and labor. The IgG monoclonal antibodies specific for the native antigen identified with these methods are suitable for in vivo therapeutic uses, but also for sandwich ELISA assays, histology, flow cytometry, immune precipitation and x-ray crystallography.

## Introduction

B cell hybridomas have been an important source of mouse monoclonal antibodies (mAbs) since the initial production in the 1970s [1]. The most common approach for producing hybridomas has been to fuse B cells from mice hyper-immunized with the antigen with a myeloma B cell line lacking hypoxanthine guanine phosphoribosyltransferase (HGPRT). The fusion mixture is then seeded into 96-well plates and the hybridomas are selected with hypoxanthine-aminopterin-thymidine (HAT) containing medium, which kills the unfused myeloma cells. Unfused B cells can’t proliferate in vitro and finally die. Only the fused hybridoma cells can survive HAT selection and divide. Most commonly, supernatants from wells showing hybridoma growth are screened for antigen-specific antibody using antigen coated ELISA plates. The cells from positive wells are then sub-cloned at limiting dilution and retested to be sure of monoclonality.

Over the years, many useful mouse monoclonal antibodies have been successfully made by this fusion and screening method. However, it has two major shortcomings. First, mice hyperimmunized with soluble proteins not only produce antibodies specific for the native protein, but also those specific for epitopes unique to the denatured protein, especially when adjuvants such as Freund’s adjuvant or alum are used in the immunization [2,3]. While these latter antibodies can be useful for detection of denatured protein in western blotting; the antibodies specific for native antigen have a much wider range of applications, including in vivo therapeutic reagents, as well as sandwich ELISA assays, histology, flow cytometry, immune precipitation and x-ray crystallography. Assays performed with antigen-coated ELISA wells generally can’t distinguish antibodies that recognize the native vs. denatured form of antigen protein, since absorption to ELISA plates denatures some of the protein due to the hydrophobic interaction between the plate surface and protein [4]. The second shortcoming is the traditional mAb selection method is labor and time consuming with several rounds of plate seeding and selection before the antibody can be fully characterized.

To overcome these shortcomings, we have developed a rapid method to characterize mouse IgG antibodies and single cell sort antigen specific IgG hybridomas cells. We captured potential antigen-specific mAbs from hybridoma culture supernatants within flow cells of a BIAcore BIAsensor chip, each containing an immobilized anti-Fc antibody specific for an individual mouse IgG isotype, with the surface plasmon resonance (SPR) signal identifying the mAb isotype. The captured mAb was tested for its ability to bind the native antigen, following the binding kinetics with SPR from which the mAb affinity was estimated. We also found that mouse hybridoma cells secreting IgG antibodies have a surface form of IgG that lacks Igα but binds antigen normally. We used fluorescent antigen bound to this surface IgG to single cell sort hybridoma cells secreting mAbs specific for native antigen.

## Material and Methods

### Materials

The Biacore CM5 chip was purchased from GE Healthcare. Goat anti-mouse IgG, Fc specific and IgGγ1, 2a, 2b and 2c specific antibodies were bought from Jackson ImmunoResearch. AKP conjugated anti-mouse IgGγ2a or IgGγ2b antibodies were from BD Pharmingen. Actin antibody was from Cell Signaling Technology. Rabbit polyclonal Igα antibody was a gift from Dr. John Cambier lab. Ovalbumin (OVA) was purchased from Sigma. Native OVA protein was obtained by dissolving OVA in PBS and collecting monomeric OVA peak from a superdex 200 size column using fast protein liquid chromatography (FPLC). Alexa fluor 647 conjugated OVA protein was purchased from Life technology. Anti-Alexa Fluor 647 MicroBeads, anti-CD43 microbeads and LS column were from Meltenyi Biotec. BCIP was from Promega. LANAC adjuvant was provided by Dr. Steven Dow. Spike protein of SARS-CoV was made by Dr. Chengyu Jiang lab.

Mice were purchased from Jackson lab and kept in a specific pathogen free environment at Naitonal Jewish Health. All mice experiments were carried out in strict accordance with the guides for the Care and Use of Laboratory Animals of National Institutes of Health. The protocol AS2517-07-13 was approved by the National Jewish Health Institutional Animal Care and Use Committee.

H22, H71 and M1 hybridoma cells against Maltose Binding Protein (MBP) were made by fusion of MBP immunized mice spleen cells with Sp2/0 in our lab. S212 hybridoma cell against the spike protein of SARS-CoV was made by Xia Hong and Janice White [5]. 2JLO-5 hybrdoma cell line against OVA was made by Laura Noges and Janice White in our laboratory. The AL1-4L3 IgGγ3 hybridoma cell line was a gift from Libuse Jerabek [6].

### Immobilization of goat anti-mouse IgG subclass antibodies on CM5 chip

Goat anti-mouse IgG, Fc subclass 1, 2a, 2b or 2c specific antibodies were immobilized in flowcells of a BIAcore CM5 as per manufacturer’s instructions. The antibodies were dissolved at 50μg/ml in 50mM sodium acetate pH5.0. The surface of CM5 was activated by N-hydroxysuccinimide (NHS)/ethyl (dimethylaminopropyl) carbodiimide (EDC). Each one of four antibodies was then injected into a different channel of a CM5 chip and the conjugation was achieved by covalent coupling of antibody to the surface of chip. Around 8000 RU of antibody was immobilized on each channel. The unoccupied activated sites of the chip were then blocked by injection of ethanolamine.

### Mice immunization

3-month old BALB/c mice were immunized intraperitoneally three times with 20 μg OVA protein mixed with LANAC (Liposomes and unmethylated DNA) with one week interval. OVA antibody titer in mouse serum was confirmed by ELISA using OVA coated plate. 20 μg OVA in PBS was then injected intraperitoneally for boosting three days before sacrificing the mice.

### Hybridoma cell surface IgG staining

Mouse hybridoma cell lines, 2JLO-5, M1, S212 and AL1-4A2 which secreted mouse IgGγ1, IgGγ2a, IgGγ2b and IgGγ3 antibodies respectively, were stained with 2 μg/ml goat anti mouse heavy chain Fc region isotype specific antibody or control antibody. Staining was carried out on ice for 15 minutes. Cell surface staining was detected by FACScan.

### Splenocytes fusion and sorting for antigen specific hybridoma cells

Splenocytes were fused with Sp2/0 myeloma cells. Instead of traditional 96-well plates, fused cells were cultured in T225 flask. HAT was added the following day. 8 days after fusion, dead cells were removed by PI staining and live cells were stained with Alexa Fluor 488 conjugated goat anti-mouse IgG antibody and Alexa Fluor 647 conjugated OVA. 2JLO-5 cell which secretes IgGγ1 antibody against native OVA was also stained as a positive control for gating purpose. Moflo XDP was used to single cell sort double positive hybridoma cells.

### RT-PCR of IgG heavy chain from hybridoma cells

mRNA from hybridoma cells was extracted in Trizol. The first stand cDNA was made using oligo-dT as primer. A common forward primer and different reverse primer targeting the end of secreted or membrane form of IgG heavy chain were used for RT-PCR using Taq polymerase. The PCR product was separated with agarose gel and verified by sequencing. The primers used were listed below in Table 1.

**Table 1:** Primers used to amplify secreted and membrane form of mouse IgG.

Primer name	Primer sequence
IgGγ1-F	GGGAATTCGAGGTGCAGCTGCAGGAGTCTGG
IgGγ1 sec-R	ACCAGGAGAGTGGGAGAGG
IgGγ1 mem-R	TTCTTGTATTCAGGAACCAGTG
IgGγ2a-F	GTCCAGTGGTGTGCACACC
IgGγ2a sec-R	CGGAGTCCGGGAGAAGCTC
IgGγ2a mem-R	TTTCTGTAGTCAGGGGAGATC
IgGγ2b-F	GGGAATTCGAGGTGCAGCTGCAGGAGTCTGG
IgGγ2b sec-R	GAGATGGTCTTCTTCAGGTAG
IgGγ2b mem-R	TTTCTGTAGTCAGGGGAGATC
IgG 3-F	CTCATCTGTCCTGCAGTCTG
IgGγ3 sec-R	GAGCGAGACAGGTTCTTCTG
IgGγ3 mem-R	GAGGAGAAGATCCACTTCACC

### ELISPOT assay

ELISA plate was coated with SARS-CoV spike protein (expressed from HEK293 cells [5]) overnight and blocked with SMEM cell culture medium containing 10% FBS. A serial dilution of S212 hybridoma cells were incubated in the wells at 37°C cell incubator for 8h. Cells were discarded and plate was washed with PBS containing 0.05% Triton X-100. AKP conjugated anti-mouse IgGγ2a or IgGγ2b antibody was then added and incubated for 1 hour at room temperature. After extensive wash with PBS containing 0.05% Triton X-100, spot was developed with BCIP in ESA buffer (100mM Tris-HCl pH9.5, 100mM NaCl and 10mM MgCl_2_) at 37°C.

### Enrichment of γ2a antibody secreting cells from γ2b antibody secreting hybridoma cells

10 million S212 hybridoma cells were stained with Alexa Fluor 647 conjugated goat anti-mouse IgGγ2a antibody, washed and then incubated with Anti-Alexa Fluor 647 MicroBeads. Microbeads bound cells retained on the LS column in the magnetic field were recovered by the removal of the magnetic field. The recovered cells were then stained with Alexa Fluor 488 conjugated goat anti-mouse IgGγ2b antibody. The Moflo XDP flow cytometry instrument was used to single cell sort IgGγ2a^+^ IgGγ2b^-^ cells from this enriched population into wells of a 96-well micro-culture plate.

### Western blotting

Hybridoma cells or CD43 bead-purified B cells were washed twice with ice-cold PBS and lysed in RIPA buffer. Purified CD43^+^ B cells were used as Igα positive control. Cell lysate was loaded on to SDS-PAGE, transferred to nitrocellulose membrane and probed with anti-Ig α antibody. Actin was also probed as a loading control.

## Results

### Using SPR to identify and characterize IgG mAbs specific for native antigen

**Fig 1:**
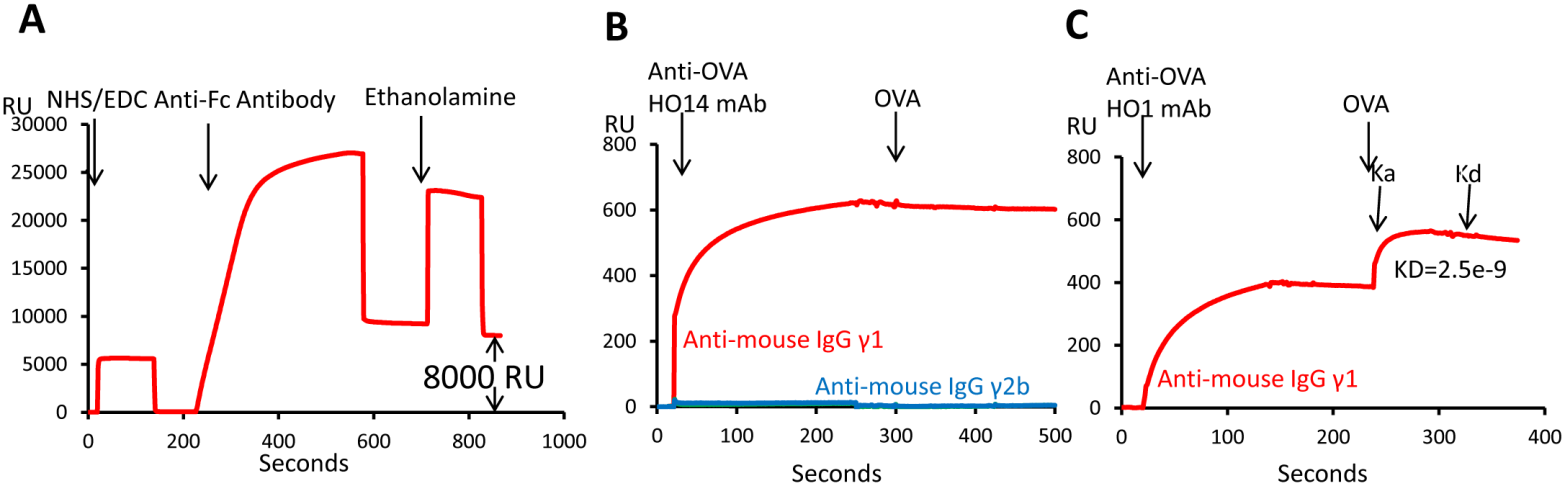
Characterization of mouse monoclonal antibodies with BIAcore. Each of four goat anti-mouse IgG Fc ɣ1, ɣ2a, ɣ2b or ɣ2c polyclonal antibodies was coated on the four different channels respectively on a CM5 chip. (A) A representative coating process of goat anti-mouse IgG ɣ1 Fc specific antibodies on the first channel of a CM5 chip. (B) Binding of IgG ɣ1 monoclonal antibody HO14 detected positive by OVA coated ELISA with native OVA protein. The blue curve represents signal of the anti-IgG ɣ2b channel subtracted by the anti-IgG ɣ2c channel. (C) Binding of IgG ɣ1 monoclonal antibody HO14 detected positive by OVA coated ELISA with native OVA protein. The dissociation rate (Kd) and association rate (Ka) between HO1 antibody and native OVA protein were calculated from the corresponding curve.

To discriminate between mAbs that bind native vs. denatured forms of protein antigens, we used Surface Plasmon Resonance (SPR) technology with a BIAcore instrument, which follows the increase in refractive index that occurs as protein binds to the surface of a flowcell [7]. We covalently immobilized four different polyclonal goat anti-mouse IgG Fc isotype specific antibodies, recognizing mouse IgGγ1, γ2a, γ2b and γ2c respectively, on the gold surface in the four different flowcells of a BIAcore CM5 BIAsensor chip (Fig 1A). These goat antibodies target the Fc region of mouse antibodies and have very high binding affinity against the mouse Fc. A hybridoma supernatant containing an IgG mAb injected through all four flowcells is captured only in the well with the appropriate anti-Fc specificity thus identifying the mAb isotype. The high affinity of the polyclonal anti-Fc reagent results in stable binding (Fig 1B). The other flowcells act as negative controls for non-specific binding and the fluid phase SPR signal from the culture supernatant.

Since the mAb is captured via its Fc region, its two Fab arms are free to interact with the antigen. By injecting the native immunizing protein, we can tell whether the captured mAb recognizes native protein. For example, HO14 and HO1 are both IgGγ1 mAbs produced from OVA-immunized mice. They were previously scored to bind to OVA protein coated on standard ELISA plates. However, when these mAbs were captured via their Fc within the anti-IgGγ1 Fc flowcell (Fig 1B and 1C), subsequently injected native OVA bound to the HO1 mAb, but not the HO14 mAb, showing that the HO14 epitope was not present on the native OVA protein. Furthermore, since OVA is a monovalent antigen, the binding kinetics, followed via the SPR signal, could be analyzed to estimate the affinity of the HO1 mAb binding to native OVA (Fig 1C). All of this analysis can be done with less than 50 μl of mAb culture supernatant.

**Fig 2:**
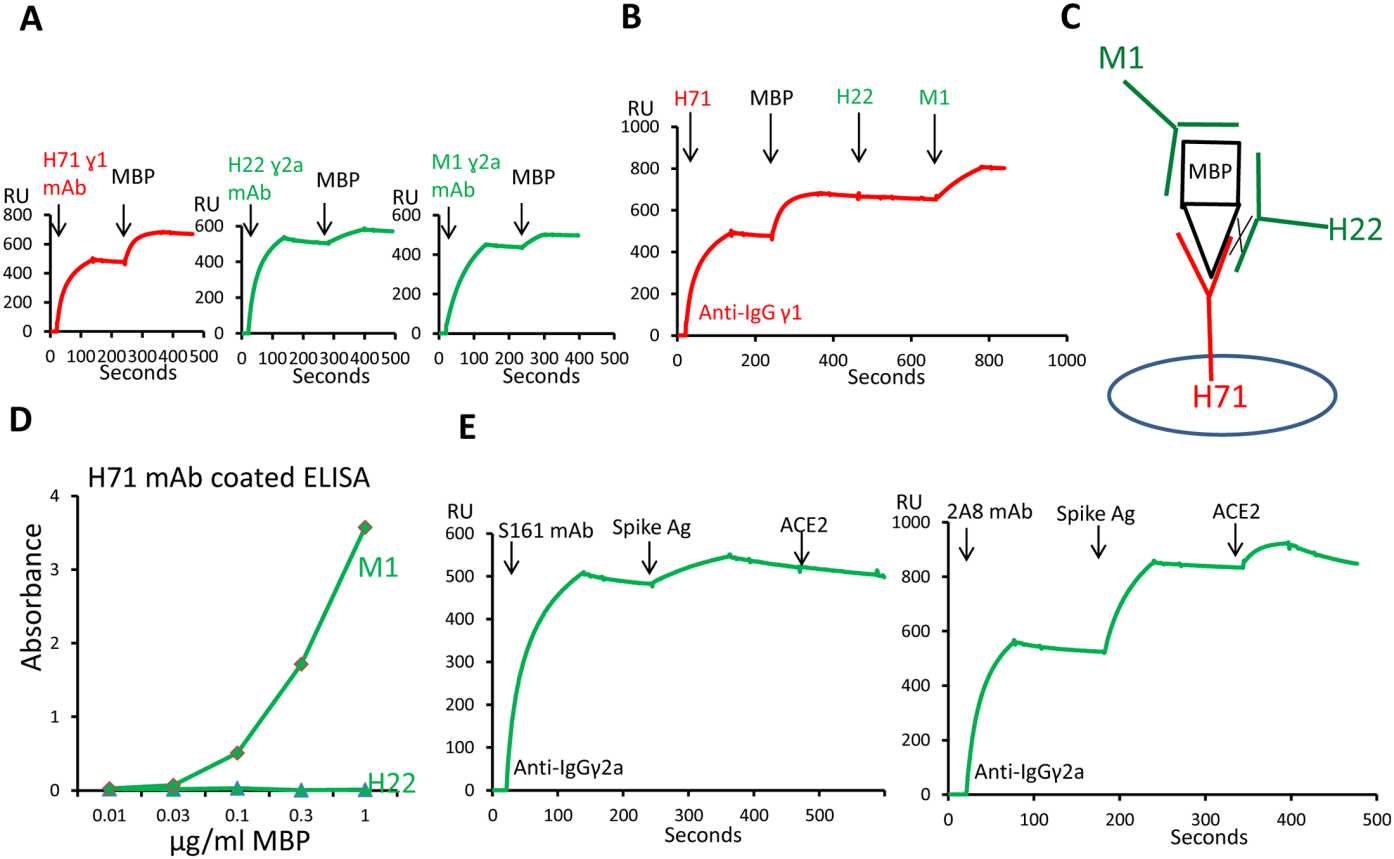
Two applications of detecting antibody-antigen interaction with BIAcore. (A) Detecting three native MBP binding antibodies separately using BIAcore. (B) Characterizing the interaction of these three antibodies with MBP using BIAcore. (C) A cartoon for the interaction of these three antibodies with MBP. (D) Pairing of MBP antibodies as coating and detection antibodies for detection of MBP. (E) Detection of interaction between two monoclonal SARS-CoV spike protein specific antibodies with spike and virus receptor ACE2.

Another useful variation of this experiment is shown in Fig 2A, 2B and 2C. Three mAbs specific for the native maltose binding protein (MBP) were characterized. H71 was an IgGγ1 mAb and H22 and M1 were IgGγ2a mAbs. We used the BIAcore system to determine whether these mAbs recognize the same or different epitopes on the antigen protein. When tested separately all three mAbs bound the native MBP (Fig 2A). To test whether these antibodies recognize different epitopes on MBP, H71 antibody was first captured on the chip and native MBP was injected and bound by H71 antibody. H22 and M1 antibody were then injected in sequence (Fig 2B). The MBP bound to the H71 mAb subsequently failed to bind the H22 mAb, indicating that its epitope was blocked by the H71 mAb. However the M1 mAb bound well to the H71 captured MBP, indicating its epitope was well separated from that of H71 (Fig 2C). This type data can allow for the rapid identification of pairs of mAbs that can be useful in developing a sandwich ELISA assay. Fig 2D shows standard sandwich ELISA data using wells coated with purified H71 mAb to capture various concentrations of MBP, which were subsequently detected with M1 mAb, but not with H22 mAb.

In vivo therapeutic uses of mAbs to block receptor-ligand interactions has been very successful. To be useful these mAbs must bind to a receptor or ligand epitope that lies within the interface of the interaction. Fig 2E shows how this SPR method can be used to identify useful mAbs of this type. S161 and 2A8 are two IgGγ2a mAb specific for the Spike protein of the SARS coronavirus (SARS-CoV). When either of these two mAbs was captured in an anti-IgGγ2a Fc flowcell, both bound injected native Spike protein. However, a subsequently injected soluble version of the Angiotensin Converting Enzyme 2 (ACE2), Spike receptor, bound to 2A8 captured Spike protein, but failed to bind to the S161 captured Spike protein. These results identify the S161 mAb as a potential therapeutic mAb for blocking the interaction of the SARS-CoV with its cellular receptor.

The robustness of the goat anti-IgG Fc reagents allows the flowcells in these experiments of be stripped of the captured mAb’s and antigen with a low pH (10mM glycine buffer, pH 1.7) wash without significant damage to the immobilized anti-Fc reagent. Thus a single BIAsensor chip can be used for hundreds of experiments.

### Correlation between surface and secreted expression of IgG in hybridomas

**Fig 3:**
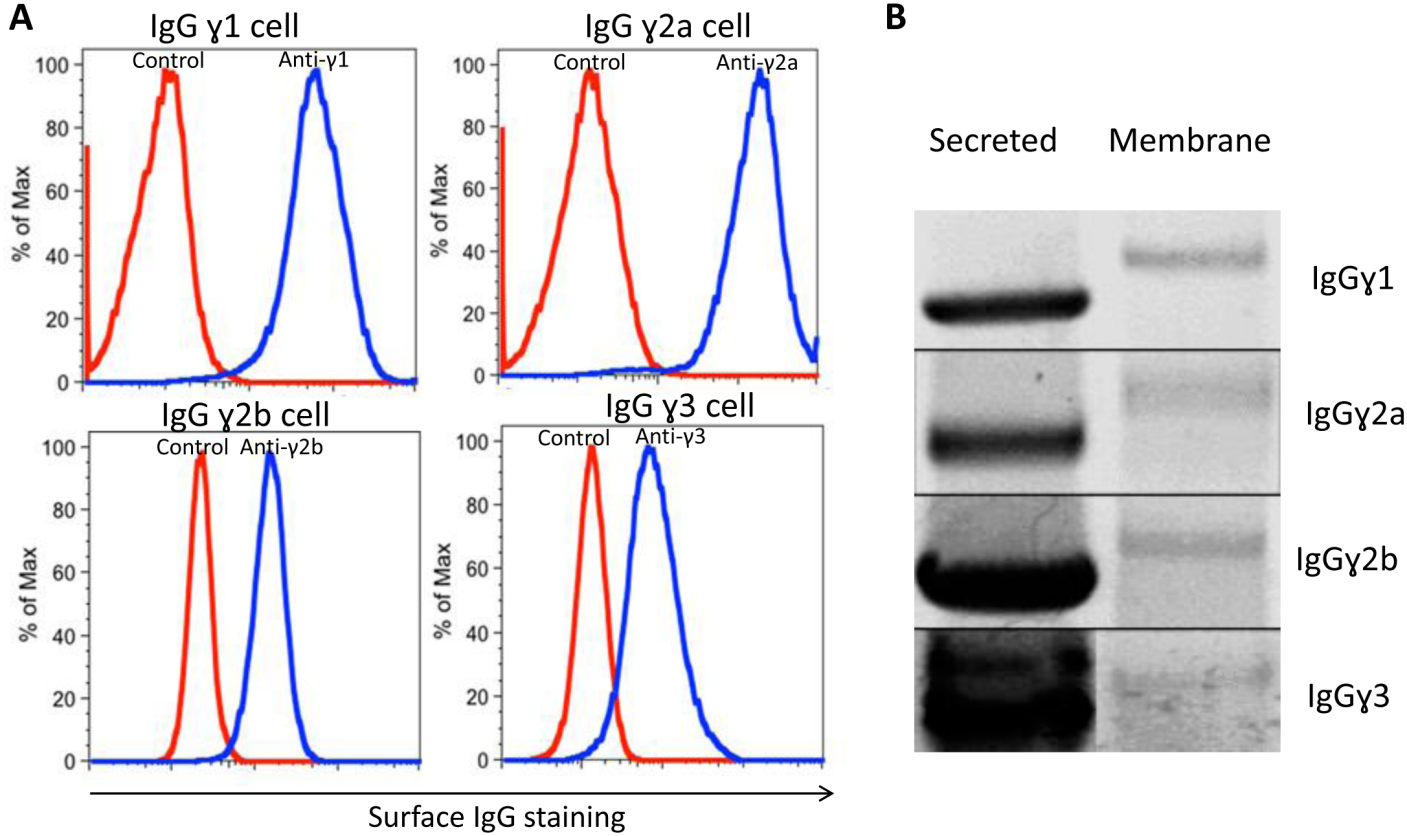
Hybridoma cells secreting mouse IgG antibodies also have surface IgG expression. (A) Staining of surface IgG expression on mouse IgG hybridoma cells with isotype specific antibodies. (B) Detection of secreted and membrane form of IgG heavy chain mRNA expression by RT-PCR.

The heavy chain secreted and membrane forms of immunoglobulins are produced from a single RNA transcript through differential splicing. In non-activated B cells this splicing results in virtually exclusive production of the membrane form. In fully differentiated antibody secreting cells the mRNA expressing the secreted form predominates, but some of surface form is still produced [8]. This has also been documented in mAb secreted B cell hybridomas [9]. We confirmed this observation in our hybridomas that were produced with the Sp2/0 myeloma fusion partner by successfully staining the cells with various fluorescent anti-IgG Fc reagents (Fig 3A). We also demonstrated the presence of the membrane form of the heavy chain mRNA in the hybridomas, although at a level much reduced from that of the secreted form (Fig 3B). Interestingly, we could not detect the expression of Igα, one of the surface IgG associated molecules involved in B cell signaling [10], in either Sp2/0 or any of our IgG producing hybridomas, suggesting that these surface IgGs are not signaling receptors (S1 Fig).

**Fig 4:**
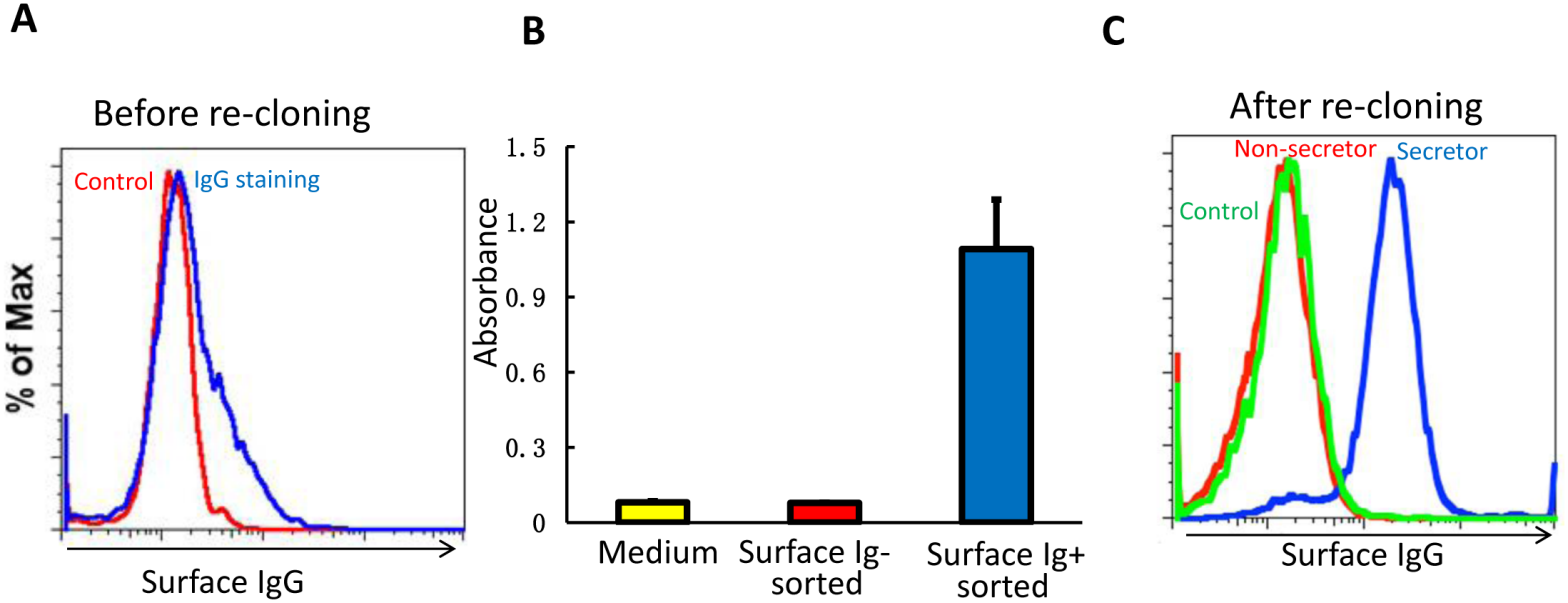
Hybridoma cell surface IgG expression correlates with antibody secretion. (A) Cell surface IgG staining of S161.1 hybridoma cell line. (B) Antibody secretion by the sorted surface IgG negative or positive cells from the S161.1 cell line. (C) Cell surface IgG staining of negative secretors or positive secretors derived from single cell colony by limiting dilution method.

We performed additional experiments to assure that there was a tight association between hybridoma cells with surface expression of IgG and those secreting IgG. A previously single cell cloned hybridoma cell line, S161, described above, began to lose IgG secretion in culture over time. We found that not all of the cells had expression of surface IgG (Fig 4A). However, after sorting the hybridoma cells with or without surface IgG expression respectively, we could only detect antibody secretion from hybridoma cells with surface IgG expression (Fig 4B). Furthermore, when the heterogeneous hybridoma cells were sub-cloned at limiting dilution, only those clones that secreted antibody had surface IgG expression (Fig 4C). This shows that the cell surface IgG expression on our Sp2/0 derived mouse hybridoma cells correlates with their ability to secrete mAbs of the same isotype. Others have suggested antigen specific hybridomas selected by cell surface Ig expression are more stable antibody secretors compared with traditional plate selected hybridomas [11].

### Isolation of IgG isotype switched variants based on surface IgG expression

For many purposes, e.g. viral protection, complement fixation, preparation of Fab fragments, the physical properties and effector functions of mouse IgGγ2a (or γ2c) mAbs are preferred over other genetically upstream isotypes. It has been reported that switching of heavy chains to downstream isotypes in mouse hybridoma cell lines occurs at an extremely low, but detectable rate, allowing the use of heavy chain isotype specific antibodies to isolate these switched variants with several rounds of selection [12–15].

**Fig 5:**
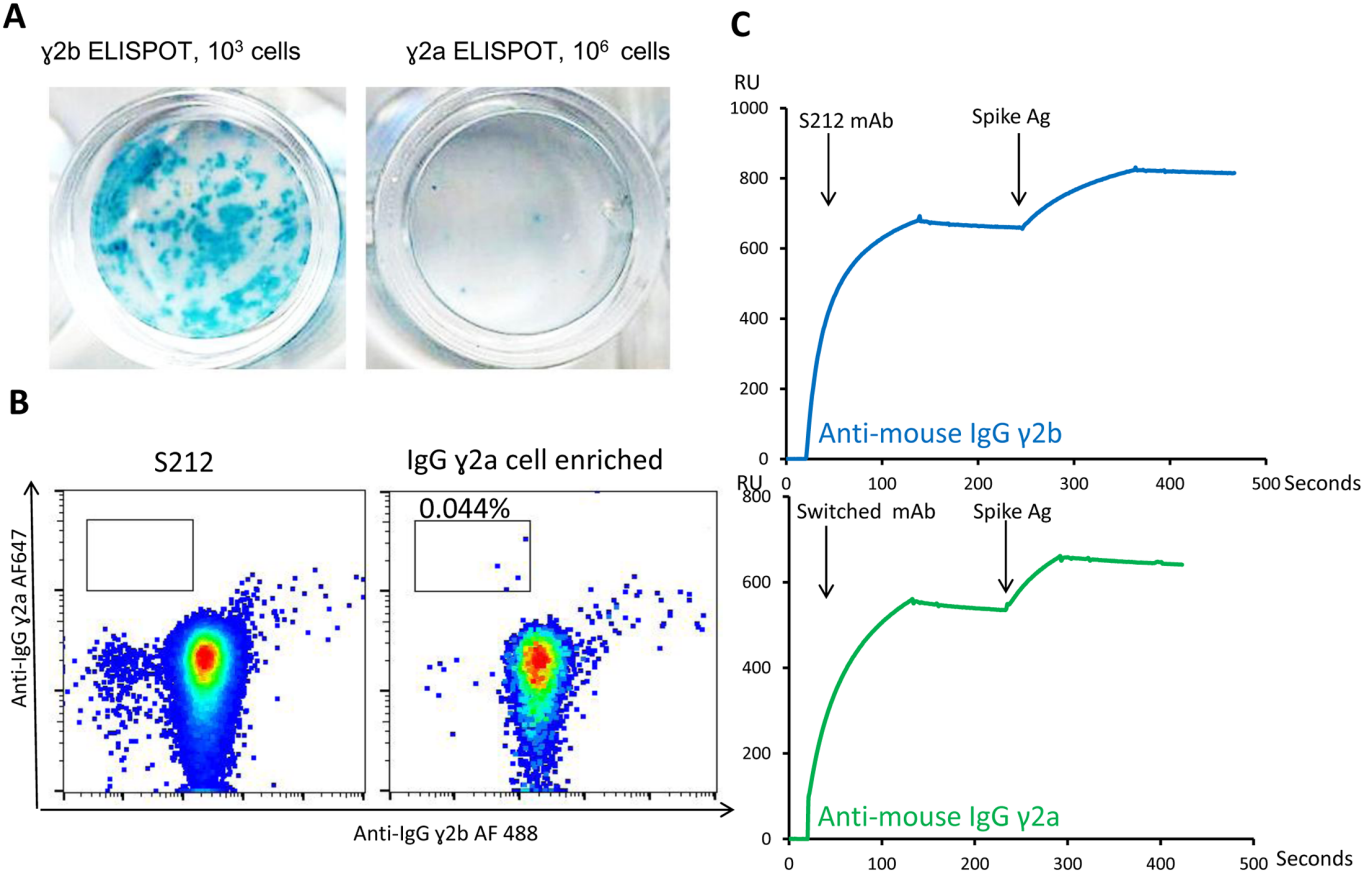
Isolation of IgG ɣ2a antibody secreting cells from an IgG ɣ2b hybridoma cell line. (A) Detection of IgG ɣ2a antibody secreting hybridoma cells in S212, an IgG ɣ2b hybridoma cell line with ELISPOT. (B) Cell surface IgG staining of S212 cells. (C) Enrichment of IgG ɣ2a secreting cells by MACS and single cell sorting of these heavy chain switched cells. (D) Biacore testing of S212 IgG ɣ2b antibody and the switched IgG ɣ2a antibody.

We used the finding that the presence of surface IgG on hybridomas correlated with their secreted IgG, to improve methods for isolating IgG class switched variants of MAbs. We started with the mouse hybridoma cell line, S212, that produced IgGγ2b mAb against the SARS-CoV Spike protein. Analyzing these cells using an ELISPOT assay, we found that IgGγ2a switched variants existed in this cell line at the rate of ~1 per 3x10^-6^/cells (Fig 5A). These cells were undetectable by direct flow cytometry, but by using an antibody specific for IgGγ2a, we were able to enrich them with magnetic beads to the point that the IgGγ2a^+^/IgGγ2b^─^ cells could now be identified with flow cytometry and single-cell sorted to produce clones (Fig 5B). Starting with 10 million S212 cells, we sorted 18 IgGγ2a cells, 12 of which grew into colonies, all of which secreted IgGγ2a instead of IgGγ2b and bound the spike protein with the same affinity as the parent S212 IgGγ2b antibody (Fig 5C). Also, they expressed the same V_H_ and V_L_ sequence as the parent antibody (Data not shown).

### Direct isolation of antigen specific hybridomas from a bulk culture of fused cells

Using SPR with initial hybridoma culture supernatants can greatly speed up the characterization of the mAbs, but the most time consuming process in making mAbs is selection and cloning hybridoma cells secreting the desired antigen-specific mAb. Normally, the initially fused cells are distributed into multiple microculture plates at a dilution estimated to produce less than one HAT resistant hybridoma per well. Since the fusion to the myeloma cells is not selective, many of the resulting HAT resistant hybridomas do not produce mAb specific for the immunizing antigen. Therefore all wells with growing hybridomas must be screened for the mAb specificity. Also, problems can arise when a miscalculation of the cell dilution results in wells with multiple HAT resistant hybridoma cells, only one of which is secreting the desired mAb. Furthermore, since after fusion initial daughter cells of the fused hybridoma cell tend to lose chromosomes, progeny in a well arise that is no longer secreting the mAb. Therefore the hybridomas cells in a positive culture well must be cloned and rescreened before the overgrowth of the desired mAb producing hybridoma cells by other HAT resistant cells in the culture well.

We performed a set of experiments to see if surface IgG on the hybridoma cells could be used to solve these problems and further reduce the time and effort to produce cloned stable clonal hybridoma cells producing only the useful mAbs. We developed a method to use fluorescent anti-mouse IgG antibodies with fluorescent antigen to single cell sort antigen-specific hybridoma cells from a fusion bulk mixture directly targeting native antigen specific hybridomas.

**Fig 6:**
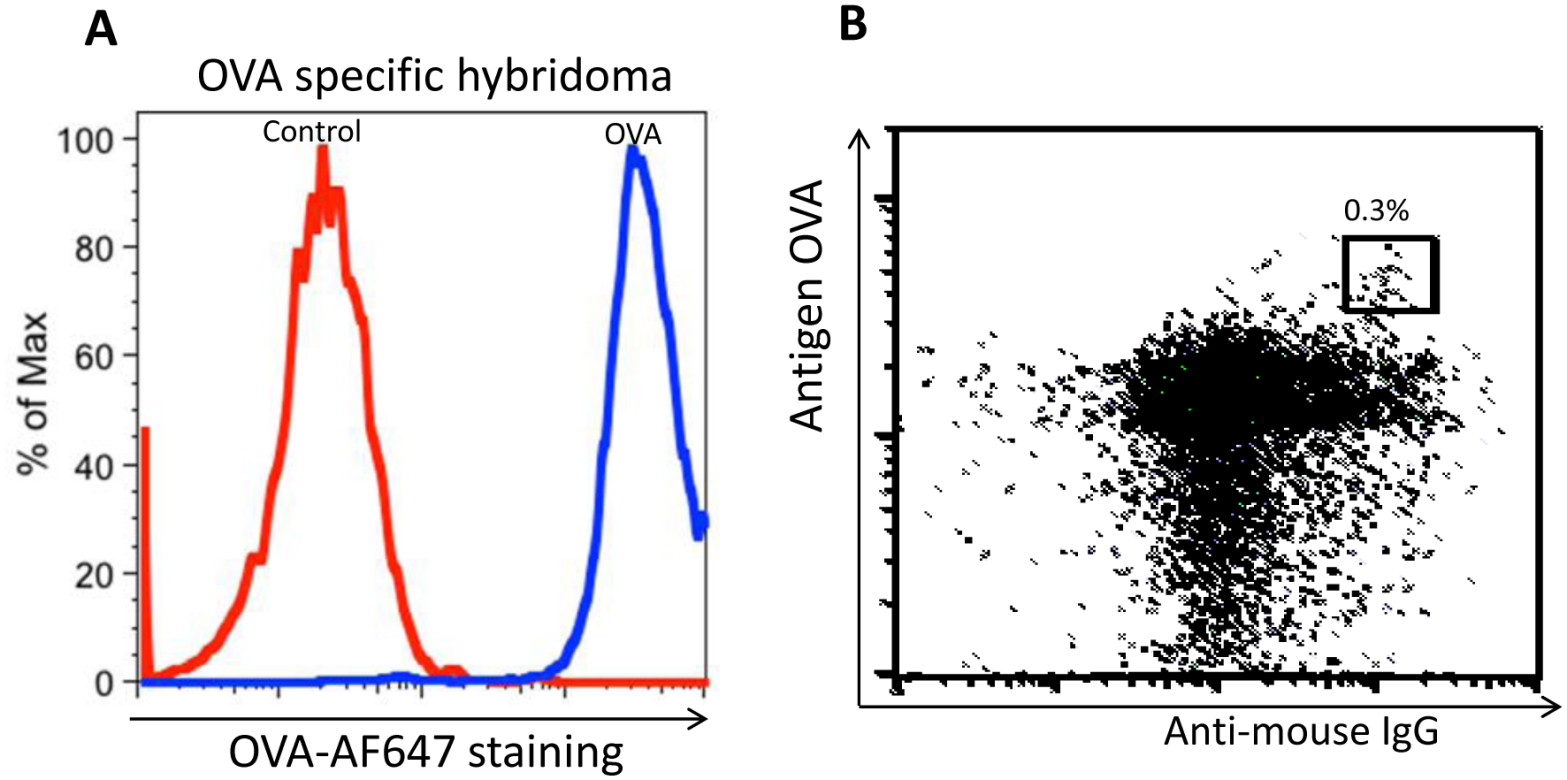
Sorting of native antigen binding IgG hybridomas from a fusion bulk mixture. (A) Staining of OVA specific hybridoma cell line with fluorescent OVA. (B) Sorting of OVA specific hybridoma clls from a fusion bulk mixture.

First we showed that fluorescently labelled OVA stained a hybridoma cell line producing an anti-OVA specific IgG mAb (Fig 6A). Then we fused spleen cells from OVA immunized mice with the Sp2/0 fusion partner in the standard way, but instead of culturing and selecting the hybridomas in microculture plates, we grew them in bulk culture with HAT for 8 days to kill the unfused Sp2/0 cells. The remaining cells were then stained with anti-IgG to identify the IgG producing hybridomas and also with the fluorescent OVA reagent to identify those specific for OVA. Cells positive for both antibodies occurred with a frequency of less 0.3% of the live cell population (Fig 6B). Single double positive cells were sorted into individual culture wells of three microculture plates and, after 10 days supernatants of the resulting colonies were tested for OVA specific IgG secretion. This selection method is efficient and accurate, as 50% of wells had hybridoma cells growing in them and nearly 90% of these secreted IgG antibodies binding the native antigen (Table 2). Since almost all of the clones secreted IgGγ1 antibody, we randomly selected 6 clones for V_H_ and V_L_ sequencing. Five of the six clones had the same V_H_ and V_L_ sequence suggesting at least some clonal outgrowth during the bulk culture (data not shown). As a negative control we also single cell sorted one plate of surface IgG positive, but OVA negative clones. All 40 of the resulting colonies secreted IgG, but only one secreted antibody binding the native OVA. This hybridoma cell was later found to be positively stained by OVA. The sorting of this hybridoma cell might be due to a system error. Thus, this selection method greatly improved the detection and selection of hybridomas producing mAb binding to the native form of the immunizing antigen prior to initial screening for antibody specificity.

**Table 2:** Summary for single hybridoma cell sorting.

	IgG ɣ1 OVA+	IgG ɣ1 OVA-	IgGɣ2a OVA+	IgG ɣ2a OVA-	IgG ɣ2b OVA+	IgG ɣ2b OVA-
IgG OVA DP^a^ plate 1	40	7	1*			
IgG OVA DP^a^ plate 2	48	5	1*			
IgG OVA DP^a^ plate 3	38	6				
IgG SP^b^ plate	1	33		3		3

^a^DP: Double positive staining by both anti-IgG antibody and OVA.

^b^SP: Single positive staining by anti-IgG antibody and negative staining by OVA.

“+” means antibody binds native OVA, while “-” means antibody does not bind native OVA in BIAcore testing.

* These two hybridomas secrete both IgG ɣ1 and ɣ2a antibodies, and both of them bind native OVA and have the same VH and VK.

## Discussion

In this paper, we optimize two methods to greatly streamline the production and characterization of mAbs, especially those specific for epitopes present on native proteins. The first uses SPR with a small amount of primary hybridoma supernatant to identify quickly the IgG isotope of the mAb, its ability to bind native antigen and its affinity for the antigen. The second uses flow cytometry to take advantage of the strong correlation between hybridoma secreted and surface IgG in order to isolate either rare cells in hybridoma cultures that have switched to useful genetically downstream isotypes or rare antigen-specific hybridomas in a heterogeneous mixture of hybridoma cells grown in bulk immediately following fusion. Taken together these methods greatly reduce the time and effort required to go from the initial fusion to the isolation of hybridoma clones producing mAbs of the appropriate specificity and functions for downstream applications.

These methods can be used to find appropriate mAbs for research, commercial and therapeutic uses. We have shown examples here of how they can be used to identify IgGγ2a switch variants, rapidly identify mAbs suitable for sandwich ELISAs and identify mAbs capable of preventing virus/receptor interactions. However, there are other potential uses as well. For example our laboratories have used these antibodies successfully to improve co-crystallization trials with the antigens that have been difficult to crystallize on their own. It is thought that these antibodies work by shielding unstructured or highly mobile regions of the proteins from disrupting internal crystal contacts [16]. For example, we have successfully co-crystallized the JMJD6 transcription factor with the Fab region of a JMJD6 specific antibody. Binding of Fab to JMJD6 is essential for crystallization as it immobilizes the C-terminal flexibility of JMJD6 [17].

Although we did not explore this use here, one could imagine how the ability to sort hybridoma cells based on the surface binding of fluorescent antigen might be used to screen IgG V-region mutant libraries in order to evolve mAbs with higher affinity IgG. These types of in vitro affinity maturation experiments are most often done via IgG surface display on phage or yeast [18]. While these systems allow for the construction of very large libraries, not all antibodies express well in these organisms [19]. This problem would be circumvented with B cell hybridoma surface expression and modern retroviral expression systems also allow for large scale mutant libraries. Moreover, the simultaneous surface expression and secretion of the mAb would make it immediately available for in vitro and in vivo functional experiments.

We and others have found that the surface form of IgG receptor on hybridomas lacks the signaling Igα co-receptor [20]. This may be of some advantage in the uses we are proposing here involving engagement of the surface IgG by antibody or antigen in order to isolate the cell. The lack of signaling through the engaged receptor may head off activation untoward downstream signaling pathway that could induce apoptosis.

## Supporting Information

S1 Fig Hybridoma cell surface IgG doesn’t form B Cell Receptor complex due to lack of Igα. Detection of Igα expression in fusion partner cell SP2/0, IgG hybridoma cells and spleen B cells by western blotting.
(TIF)

S1 Document Experimental details for the animal work. (DOCX)
