# Supplementary figures and images for: A Rapid Method to Characterize Mouse IgG Antibodies and Isolate Native Antigen Binding IgG B Cell Hybridomas
Source: PLoS One. 2015 Aug 28;10(8):e0136613. doi: 10.1371/journal.pone.0136613 (PMC4552657; doi:10.1371/journal.pone.0136613)

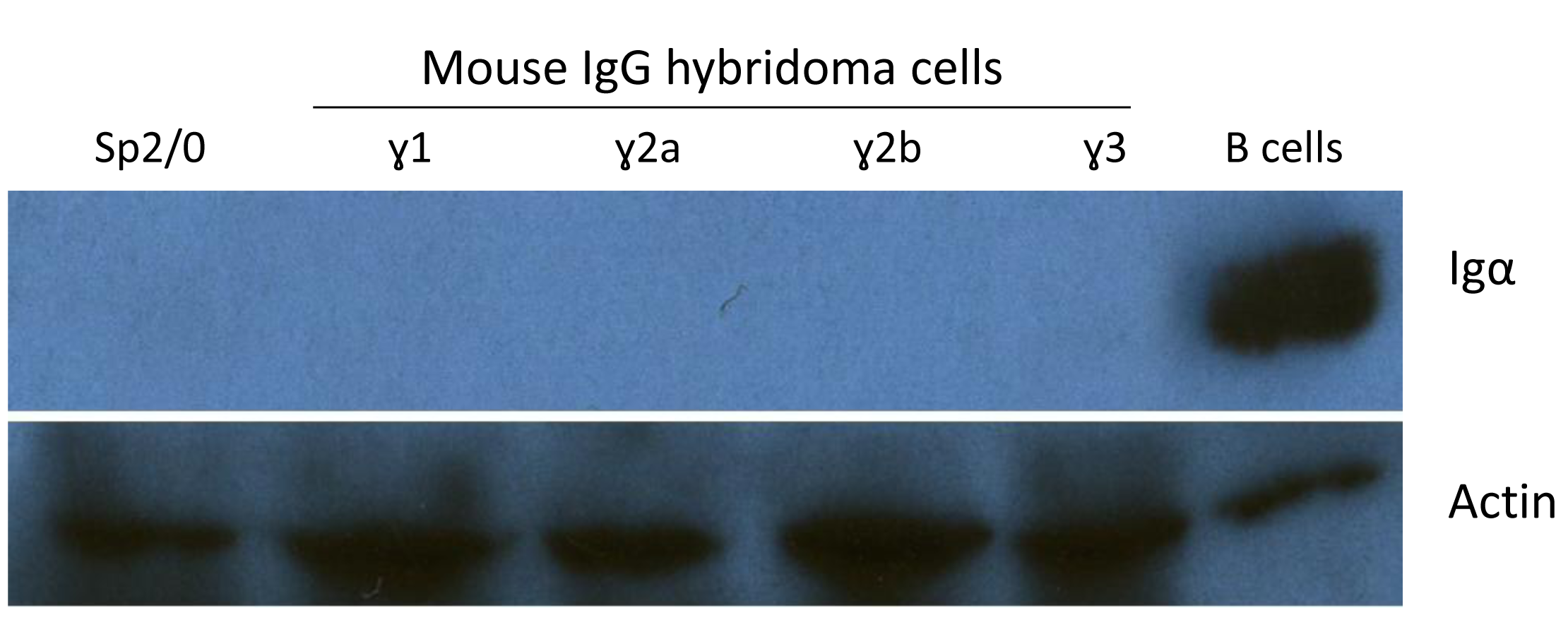

Supplement: S1 Fig — Detection of Igα expression in fusion partner cell SP2/0, IgG hybridoma cells and spleen B cells by western blotting. (TIF) [file pone.0136613.s001.tif]
