# Supplementary material for: A Rapid Method to Characterize Mouse IgG Antibodies and Isolate Native Antigen Binding IgG B Cell Hybridomas
Source: PLoS One. 2015 Aug 28;10(8):e0136613. doi: 10.1371/journal.pone.0136613 (PMC4552657; doi:10.1371/journal.pone.0136613)
Supplement: S1 Document — (DOCX) [file pone.0136613.s002.docx]

S2. Experimental details for the animal work.

| Title 1 Provide as accurate and concise a description of the content of the article as possible.   \|  \|  \|  \| \| --- \| --- \| --- \| | Immunization of mice with OVA protein and fusion of splenocytes with Sp2/0 myeloma cells to make OVA specific hybridoma cells. Native OVA specific hybridoma cells were then sorted by single cell sorting based on surface IgG expression instead of traditional limiting dilution method. |
| --- | --- | --- | --- | --- |
| Abstract 2 Provide an accurate summary of the background, research objectives, including details of the species or strain of animal used, key methods, principal findings and conclusions of the study.   \|  \|  \|  \| \| --- \| --- \| --- \| | 3-month old female BALB/c mice were immunized intraperitoneally three times with OVA protein mixed with LANAC (Liposomes and unmethylated DNA) with one week interval. OVA in PBS was then injected intraperitoneally for boosting three days before sacrificing the mice. Mice were sacrificed by cervical dislocation and spleen was removed. Splenocytes were fused with Sp2/0 cells to make OVA specific hybridoma cells using PEG8000 as fusion agent. Instead of traditional culturing fused cells in 96-well plate, we cultured them in a flask, and used fluorescent OVA to sort native antigen specific cells by single cell sorting. |
| \| Background \| 3 \| Include sufficient scientific background (including relevant references to previous work) to understand the motivation and context for the study, and explain the experimental approach and rationale. \| \| --- \| --- \| --- \| | The traditional monoclonal antibody selection method is labor and time consuming with several rounds of plate seeding and selection before the antibody can be fully characterized. We found that IgG hybridoma cells also have surface IgG expression without Igα, which could be used to sort native antigen specific hybridoma cells by single cell sorting. |
| Explain how and why the animal species and model being used can address the scientific objectives and, where appropriate, the study’s relevance to human biology. | For a long time, BALB/c mice have been immunized to provide antigen specific B cells, fusion of which with myeloma partner cells would form hybridoma cells. However, the traditional method to isolate antigen specific hybridoma cells is time and labor consuming. We set up a rapid method to isolate these cells. |
| \| Objectives \| 4 \| Clearly describe the primary and any secondary objectives of the study, or specific hypotheses being tested. \| \| --- \| --- \| --- \| | The primary objective of this study is to sort the native antigen binding hybridoma cells directly from fusion mixture, which greatly saves time and labor compared with the traditional method. |
| \| Ethical statement \| 5 \| Indicate the nature of the ethical review permissions, relevant licences (e.g. Animal [Scientific Procedures] Act 1986), and national or institutional guidelines for the care and use of animals, that cover the research. \| \| --- \| --- \| --- \| | All animal experiments were carried out at National Jewish Health in strict accordance with the Guides for the Care and Use of Laboratory Animals of the National Institutes of Health. The protocol was approved by the National Jewish Health Institutional Animal Care and Use Committee (IACUC), protocol number: AS2517-07-13. Mice were maintained in a specific-pathogen-free environment. |
| \| Study design \| 6 \| For each experiment, give brief details of the study design including:  The number of experimental and control groups.  Any steps taken to minimise the effects of subjective bias when allocating animals to treatment (e.g. randomisation procedure) and when assessing results (e.g. if done, describe who was blinded and when).  The experimental unit (e.g. a single animal, group or cage of animals).  A time-line diagram or flow chart can be useful to illustrate how complex study designs were carried out. \| \| --- \| --- \| --- \| | There were five mice in the immunization group and each of them was immunized three times with OVA/LANAC with one week interval as stated in the materials and methods in the text. |
| \| Experimental procedures \| 7 \| For each experiment and each experimental group, including controls, provide precise details of all procedures carried out. For example: \| \| --- \| --- \| --- \| \| a. How (e.g. drug formulation and dose, site and route of administration, anaesthesia and analgesia used [including monitoring], surgical procedure, method of euthanasia). Provide details of any specialist equipment used, including supplier(s).  b. When (e.g. time of day).  c. Where (e.g. home cage, laboratory, water maze).  d. Why (e.g. rationale for choice of specific anaesthetic, route of administration, drug dose used). \| \| \| | 20 μg OVA protein was mixed with 10 μl DOTIM liposome and 3μl empty vector DNA and the mixture was injected intraperitoneally to each mouse. Mice were injected three times with one week interval. OVA in PBS was then injected intraperitoneally for boosting three days before sacrificing the mice. Mice were sacrificed by cervical dislocation and spleen was removed for fusion. |
| \| Experimental animals \| 8 \| a. Provide details of the animals used, including species, strain, sex, developmental stage (e.g. mean or median age plus age range) and weight (e.g. mean or median weight plus weight range).  b. Provide further relevant information such as the source of animals, international strain nomenclature, genetic modification status (e.g. knock-out or transgenic), genotype, health/immune status, drug or test naïve, previous procedures, etc. \| \| --- \| --- \| --- \| | Three month old female BALB/c mice were purchased from the Jackson laboratory. |
| \| Housing and husbandry \| 9 \| Provide details of:  a. Housing (type of facility e.g. specific pathogen free [SPF]; type of cage or housing; bedding material; number of cage companions; tank shape and material etc. for fish). \| \| --- \| --- \| --- \|   b. Husbandry conditions (e.g. breeding programme, light/dark cycle, temperature, quality of water etc for fish, type of food, access to food and water, environmental enrichment).  c. Welfare-related assessments and interventions that were carried out prior to, during, or after the experiment. | Mice were maintained in specific-pathogen-free environment at National Jewish Health animal facility. 12-hour day/night cycle was used. All mice had free access to water and food and the cage had wood shaving and bedding. |
| \| Sample size \| 10 \| a. Specify the total number of animals used in each experiment, and the number of animals in each experimental group.  b. Explain how the number of animals was arrived at. Provide details of any sample size calculation used.  c. Indicate the number of independent replications of each experiment, if relevant \| \| --- \| --- \| --- \| | All five mice were used at different days for fusion. The data in the text was representative from one mouse. |
| \| Allocating animals to experimental groups \| 11 \| a. Give full details of how animals were allocated to experimental groups, including randomisation or matching if done.  b. Describe the order in which the animals in the different experimental groups were treated and assessed. \| \| --- \| --- \| --- \| | There was no comparation done between mice. |
| \| Experimental outcomes \| 12 \| Clearly define the primary and secondary experimental outcomes assessed (e.g. cell death, molecular markers, behavioural changes). \| \| --- \| --- \| --- \| | We harvested more or less the same number of splenocytes from all the five mice after immunization. |
| \| Statistical methods \| 13 \| a. Provide details of the statistical methods used for each analysis.  b. Specify the unit of analysis for each dataset (e.g. single animal, group of animals, single neuron).  c. Describe any methods used to assess whether the data met the assumptions of the statistical approach. \| \| --- \| --- \| --- \| | No statistical was done between mice. |
| \| Baseline data \| 14 \| For each experimental group, report relevant characteristics and health status of animals (e.g. weight, microbiological status, and drug or test naïve) prior to treatment or testing. (This information can often be tabulated). \| \| --- \| --- \| --- \| | All mice were healthy during the immunization process. |
| \| Numbers analysed \| 15 \| a.Report the number of animals in each group included in each analysis. Report absolute numbers (e.g. 10/20, not 50%).  b. If any animals or data were not included in the analysis, explain why. \| \| --- \| --- \| --- \| | Mice were not divided into groups. All of them were used and sacrificed at different days. |
| \| Outcomes and estimation \| 16 \| Report the results for each analysis carried out, with a measure of precision (e.g. standard error or confidence interval). \| \| --- \| --- \| --- \| | All mice had high titers of OVA antibodies in the serum. After fusion, we got OVA specific hybridomas. |
| \| Adverse events \| 17 \| a. Give details of all important adverse events in each experimental group.  b. Describe any modifications to the experimental protocols made to reduce adverse events. \| \| --- \| --- \| --- \| | No adverse events were observed during the immunization process. |
| \| Interpretation/ scientific implications \| 18 \| a. Interpret the results, taking into account the study objectives and hypotheses, current theory and other relevant studies in the literature.  b. Comment on the study limitations including any potential sources of bias, any limitations of the animal model, and the imprecision associated with the results.  c. Describe any implications of your experimental methods or findings for the replacement, refinement or reduction (the 3Rs) of the use of animals in research. \| \| --- \| --- \| --- \| | Since mouse IgG hybridoma cells also have surface IgG expression and due to the fact that membrane form IgG do not need Igα and Igβ for surface expression because of the composition of amino acids in the transmembrane region, it is possible that IgG hybridoma cells from other sources, such as rat and hamster, also have surface IgG expression, which can be used to isolate native antigen specific hybridoma cells. |
| \| Generalisability/ translation \| 19 \| Comment on whether, and how, the findings of this study are likely to translate to other species or systems, including any relevance to human biology. \| \| --- \| --- \| --- \| | This finding of hybridoma surface IgG expression could also be true for other species. |
| \| Funding \| 20 \| List all funding sources (including grant number) and the role of the funder(s) in the study. \| \| --- \| --- \| --- \| | This work was supported by Howard Hughes Medical Institute to John Kappler, who conceived this study. |
